# Cadmium Contaminants in Pollen and Nectar Are Variably Linked to the Growth and Foraging Behaviors of Honey Bees

**DOI:** 10.3390/insects16030306

**Published:** 2025-03-15

**Authors:** Dawei Li, Jia Liu, Yibin Yuan, Juanli Chen, Junpeng Mu

**Affiliations:** 1 Ecological Security and Protection Key Laboratory of Sichuan Province, Mianyang Normal University, Mianyang 621000, China; ldw0217@163.com (D.L.); 18272620657@163.com (J.L.); juanlic@163.com (J.C.); 2 Chengdu Academy of Environmental Science, Chengdu 610072, China; yuanyb@cdaes.org.cn

**Keywords:** *Apis mellifera*, environment pollution, plant–pollinator interactions, pollinator decline, oil-seed rape

## Abstract

Cadmium (Cd) is a heavy metal present in pollen and nectar that affects pollinator attributes. Honey bees possess the ability to eliminate Cd from honey. Consequently, the concentration of Cd in pollen, rather than nectar, is the critical factor influencing the growth and foraging behavior of honey bees. However, there is a dearth of studies regarding the specific association by which the impact on bee growth and foraging behavior fluctuates in relation to the Cd dosage of pollen or nectar. We hypothesized that at low exposure levels, the amount of Cd in pollen would affect honey bee growth, and the amount of nectar influences honey bee foraging behavior. At high exposure levels, the amount of Cd in pollen and nectar would affect both honey bee growth and foraging behavior. A field experiment was performed in Sichuan (the average background value of Cd in soil is 5.6 times higher than other regions in China) to examine the impact of different soil Cd concentrations (low: 0.60 ± 0.05 mg·kg^−1^ (average ± SD); middle: 1.32 ± 0.08 mg·kg^−1^; high: 1.76 ± 0.10 mg·kg^−1^) on the Cd levels in plant organs (*Brassica campestris*), alongside the body mass and visitation rates of honey bees (*Apis mellifera*). Our results indicated in honey bees in the habitats with low concentrations of soil Cd that the Cd content in pollen was inversely correlated with the body mass of larvae, pupae, and worker bees. The quantity of nectar governed the foraging activity of honey bees in the habitats with low levels of soil Cd. At middle to high exposure levels, Cd concentrations in pollen and honey exerted a negative influence on honey bee development and foraging behavior. These findings offer novel insights into the impact of Cd on pollinator attributes, and the global decline of pollinators.

## Simple Summary

There is a global decline in the richness of pollinators. Cadmium, a widespread contaminant, has a significant impact on pollinator life cycle, richness, and abundance. Recent research has demonstrated that honey bees possess the ability to eliminate Cd from honey; consequently, the concentration of Cd in pollen, rather than nectar, is the critical factor influencing the growth and foraging behavior of honey bees. A field experiment was performed in Sichuan to examine the impact of different soil Cd concentrations on the Cd levels in plant organs, alongside the body mass and visitation rates of honey bees. Our findings showed that in honey bee habitats with low levels of Cd in the soil, the amount of Cd in the pollen was negatively related to the body mass of the larvae, pupae, and worker bees. The quantity of nectar governed the foraging activity of honey bees in the habitats with low levels of soil Cd. At middle to high exposure levels, Cd concentrations in pollen and honey exerted a negative influence on honey bee development and foraging behavior.

## 1. Introduction

Environmental contamination has profound consequences for ecosystem processes, activities, and services, as well as human health [1,2,3]. Heavy metals infiltrate both terrestrial and aquatic ecosystems, impacting the element flow, the diversity and population of flora and fauna, and even ecosystem security [4,5,6,7]. For example, lead (Pb) moves to the soil, where it then enters the roots of crops, accumulating significant amounts of Pb within the stems, leaves, and grains [8]. The consumption of substances with elevated heavy metal concentrations by an organism might result in immune system suppression, weight loss, and potentially lethal outcomes. Mercury infiltrates aquatic habitats and builds up to significant amounts in fish [9]. Consuming fish that contain high amounts of mercury poses a significant health risk to people. Cadmium (Cd) is a common heavy metal that is mostly present in its natural form in the soil and lithosphere. Human activities have also led to the buildup of Cd in the atmosphere, water, and soil [10,11]. The root uptakes Cd from the soil and concentrates it in various plant tissues, with a particular affinity for the reproductive organs. When Cd accumulates in pollen and nectar, it poses a possible risk to the safety of pollinators, particularly bees [12,13].

There is a global decline in the richness of pollinators [14]. Evidence shows that the percentage of managed honey bee colonies decreased by 43% in the US between 2019 and 2020 [15]. Various factors influence the richness and abundance of pollinators [16]. Cadmium, a widespread contaminant, has a significant impact on pollinator life cycle, richness, and abundance [6,12,17,18]. For example, the accumulation of Cd in pollen and nectar hinders bee larvae’s normal growth and interferes with their usual transition into pupae [12,17,19]. Several studies have extensively shown the effects of Cd on different aspects of pollinating insects, such as the development, lifespan, gut microbiota, brain networks, and cognition of bumble bees and honey bees [12,13,20].

Plants that thrive in soil with low levels of cadmium only transmit a small quantity of Cd to their pollen and nectar. However, pollen contains a substantially higher amount of Cd compared to honey, because bees can partially purify nectar by removing heavy metals during honey production [21]. For example, the Cd and Pb content in honey is decreased by 75% and 73% compared to that in the nectar [22]. Bogdanov found that metal contamination levels are lower in honey compared to those in the bodies of bees [23]. Thus, rather than the honey, the level of Cd in pollen is the decisive component for the development of bees. Moreover, when exposed to low levels of soil Cd, the amount of nectar influences bees’ foraging behavior [24]. At high levels of exposure, bees are unable to effectively eliminate the substantial amounts of this poisonous chemical present in the nectar [21]. As a result, cadmium accumulates in both pollen and honey. This mutually impacts bee growth and foraging behavior. For example, Gekière found that high concentrations of Cd induce severe gut melanisation of bumble bees and reduced sucrose consumption [25]. Once the pollen and honey exceed a specific Cd threshold, larvae’s growth and pupation are hampered [12,17]. However, there is a dearth of studies regarding the specific association by which the impact on bee growth and foraging behavior fluctuates in relation to the Cd dosage of pollen or nectar. Herein, we speculate that the relationships by which soil cadmium affects bee growth and foraging behavior vary with the dose of cadmium in the soil. The soil in Sichuan has a significantly elevated Cd concentration compared to other regions in China. In Sichuan, the average background value of cadmium is 5.6 times higher than in other regions due to the parent materials, mining, and industrial activities [26]. Herein, we test the effects of low or high soil Cd concentrations on the honey bee growth and foraging behavior in Sichuan, China. Our findings shed a new light on the effects of Cd on pollinator growth and foraging behavior and the global decline of pollinators.

## 2. Materials and Methods

### 2.1. Study Site

This study was conducted in Mianyang City, Sichuan Province, China (30°42′–31°42′ N, 103°54′–104°60′ E). The climate is a subtropical humid monsoon with hot summers and cold winters. The annual mean temperature is 14.9–16.8 °C [27]. The annual mean precipitation is 546–1237 mm [28]. The vegetation is dominated by broad-leaved forest, coniferous forest, and low shrubs.

Three rehabilitation zones of decommissioned battery facilities were the focus of the investigation. The irrigation of wastewater from these facilities has contaminated the land near the battery companies with cadmium. The proximity to the factory area correlates with an increased Cd concentration in the soil. This study involved the selection of three transects. Soil Cd levels at 1, 3, and 7 km from decommissioned battery facilities were 1.68 mg·kg^−1^, 1.33 mg·kg^−1^, and 0.59 mg·kg^−1^ in transect 1; 1.65 mg·kg^−1^, 1.31 mg·kg^−1^, and 0.60 mg·kg^−1^ in transect 2; and 1.66 mg·kg^−1^, 1.33 mg·kg^−1^, and 0.61 mg·kg^−1^ in transect 3, respectively (Figure S1). Here, we defined the low, middle and high levels of soil Cd as 0.60 ± 0.05 mg·kg^−1^, 1.32 ± 0.08 mg·kg^−1^, and 1.76 ± 0.10 mg·kg^−1^, respectively. At each transect, ten plots (15 m × 20 m) were designated on the outer perimeter of concentric rings (Figure S1). There were 3 sites, and 90 plots in total.

Oil-seed rape (*B. campestris*) is an annual herbaceous species. It is self-incompatible and distributed worldwide [29]. In this study, the sample species is Deyou 5 (a variety of the species *B. campestris*, made in Mianyang Special Research Seed Industry Co., Ltd., Mianyang, China), which is a local cultivar. This cultivar is classified as a medium-maturity two-line hybrid cabbage. Plant height is around 150–200 cm and features green stems, wide and green leaves, visible wrinkles and serrations on the leaf edges, and a substantial amount of wax powder on the leaf stems. The branching pattern consists of 9–11 core branches and 5 secondary branches, resulting in a consistent distribution of branches.

### 2.2. Experimental Design

Plant pots were sterilized with 70% ethanol. They were then filled with Pindstrup Substrate (Pindstrup substrate No. 5, Pindstrup Mosebrug A/S, AUSTRALMEDIA Ltda., Australia, Cd content of 0 mg·kg^−1^, see [30]). In mid-September 2020, we planted two seeds in each pot, positioned the pots within a greenhouse, and linked them to a drip water irrigation system.

In late September 2020, each plot (Figure S1) was fertilized with 24.75 kg urea, 26.25 kg superphosphate, and 11.25 kg potassium sulfate. In early October 2020, the seedlings were transplanted into the plots. Each plot had 2000 seedlings. In total, there were 90 plots and 180,000 seedlings. In late February 2021, we constructed a bamboo framework and enclosed each plot with screens (2.0 mm), ensuring that the framework had a height of 2.5 m. In early March 2021, we put the hives in the plots. Each plot contained a solitary hive with six frames, three of which were devoid of honey production, eggs, larvae, and pupae prior to the experiment. Each solitary hive harbored a population of 10,000–15,000 worker bees, 1 queen, and 10 to 30 drones [31]. The worker bees were of the same age.

### 2.3. Measurements

From March to May 2021, we assessed plant traits, body mass, honey bee visitation rates, soil cadmium levels, soil pH, and soil nutritional content. During the peak flowering period, the traits were assessed for each plot, including nectar volume per flower, nectar concentration, the Cd amounts of pollen and nectar, roots, leaves, stems, flowers, honey, worker bees, the body mass of larvae, pupae, and worker bees, as well as pollinator visitation rates. At the end of the experiment, we measured the Cd levels of soil, along with its pH, total nitrogen (TN), total phosphorus (TP), and total potassium (TK) levels.

We randomly selected 3 plants from each plot and 3–5 flowers from each plant. Next, we covered each flower with cylindrical metal netting for 24 h to keep pollinators away before measuring the nectar volume and concentration per flower [32]. Nectar volume per flower was measured using 1 or 2 µL micropipettes. At the same time, a hand refractometer with a precision of 0.5% was used to monitor nectar concentration (Eclipse, Stanley and Bellingham, Basingstoke, UK). 

To measure the Cd content of nectar, pollen, and honey, we collected the samples. Nectar collection was performed using the centrifuge [33]. Nectar was collected for each plot and subsequently placed into a designated tube. Meanwhile, we placed a pollen trap in each hive and collected the pollen grains. Thereafter, we also collected 2 mL of honey from each hive. Notice that the honey was harvested exclusively from the frames containing honey generated by the honey bees during the experiment (i.e., three frames were devoid of honey production before the experiment). There were 2 mL of nectar and 2 g of pollen for each plot.

Each of the plants were harvested individually and divided into root, stem, leaf, and flower [34]. The harvested plant materials were finally dried at 65 °C in an oven for 48 h.

We performed a random selection of 10 larvae, pupae, and worker bees from every beehive. The samples were obtained from the frames that lacked larvae prior to the experiment. The larvae, pupae, and worker bees were assessed on the 7th, 16th, and 21st days following the queen bee’s initial oviposition during the peak flowering phenology [35]. The body mass was determined using a balance with a precision of 0.001 g. The sample collection followed the protocols of Zoltowska et al. [36].

We measured the pollinator visitation rate to reflect the foraging behavior of honey bees. Pollinator visitation rate was defined as the number of pollinator visits per flower per hour, following the protocols of Arroyo et al. [37] and Vaudo et al. [38]. During the peak flowering periods, we randomly selected 5 plants per plot to monitor the visitation rates of honey bees (*A. mellifera*). Then, we recorded the number of visits to the flowers of each plant (only for flowers that opened). On each of the nine sunny days, honey bees were observed hourly for 2 min at a distance of 3 m from 09:00 to 17:00 h. Each plant was observed simultaneously for 1 min every hour. A total of 144 min of field observations were recorded for each plant. Pollinator visitation rate (R_V/F_; flower^−1^·h^−1^) was calculated as the total number of visits (N_V_) divided by the number of flowers (N_F_), i.e., R_V/F_ = N_V_/N_F_.

After collecting all the plant samples, soil samples were obtained within the depth range of 0 to 20 cm using a soil auger with a diameter of 20 cm. The soil samples were carried to the lab. The soil pH was assessed using a Soil All-in-One Soil Meter, while the determination of TN, TP, and TK was conducted through the utilization of an elemental analyzer, sulfuric acid–perchloric acid digestion method [39], and the flame photometer [40]. The Cd content of soil, pollen, nectar, honey, and worker bees was measured employing the graphite furnace atomic absorption spectrophotometry [41].

### 2.4. Data Analyses

In this study, all data on each trait were averaged for each plot before being analyzed. We performed all statistical analyses using R 4.4.1. Using a Wilcoxon rank sum test, we compared the Cd levels of soil, roots, leaves, stems, flowers, pollen, nectar, honey, and worker bees, as well as the body mass of larvae, pupae, and worker bees, pollinator visitation rate, nectar volume, and concentration. We used generalized linear mixed models (GLMMs) to look at how the amount of Cd in the soil and its properties affected plant traits, bee body mass, and how they foraged. The fixed effects encompassed soil Cd treatments (low, middle, and high levels of Cd) and the soil properties (TN, TP, TK, pH), while the plot was considered a random effect [42]. We conducted random forest analysis by using the “randomForest” package to identify key factors influencing the body mass of larvae, pupae, and worker bees, as well as pollinator visitation rates [43]. The number of trees was 500. To explore the contribution of the factors, we conducted a linear mixed-effects model analysis based on the “glmm.hp” package [44]. Using the R package ‘linkET’, we examined correlations across all the variables [45]. Relationships between pollinator visitation rate and flow rewards, body mass and flower rewards, the Cd content of worker bees, and flower rewards were determined with regression curves, and the above-mentioned parameters were fitted using the method of least squares.

## 3. Results

### 3.1. Effects of Soil Cd Concentration on Plant Traits

The quantity of cadmium in the soil, as opposed to total nitrogen, total phosphorus, total potassium, and pH, significantly affected the Cd levels in various plant parts, including leaves, flowers, stems, roots, pollen, nectar, and honey (Table 1, Table 2 and Table 3).

As the soil Cd concentrations increased, there was a considerable increase in Cd amounts of plant organs (Figures S2 and S3). Compared to low concentrations of soil Cd treatments, the amounts of Cd in leaves, flowers, stems, roots, pollen, nectar, and honey increased by 20.85%, 46.65%, 54.69%, 57.37%, 50.91%, 26.92%, and 230.77% in middle concentrations of soil Cd treatments. Similarly, at high concentrations of soil Cd treatments, the Cd amounts in these plant parts increased by 46.07%, 75.98%, 88.57%, 132.60%, 133.64%, 84.62%, and 484.62%, respectively. However, as the soil Cd concentration increased, the nectar volume per flower diminished, but nectar concentration increased (Figure S3). Through the course of the experiment, it was determined that the concentration of Cd in the nectar was 0.026 ± 0.004 mg·kg^−1^ under conditions of low soil Cd concentration. Nevertheless, the honey’s Cd concentration was measured at 0.013 ± 0.006 mg·kg^−1^, and it decreased by 50% (Figure S3).

### 3.2. Effects of Soil Cd Concentration on Body Mass and Cd Concentration of Honey Bees

**Figure 1:**
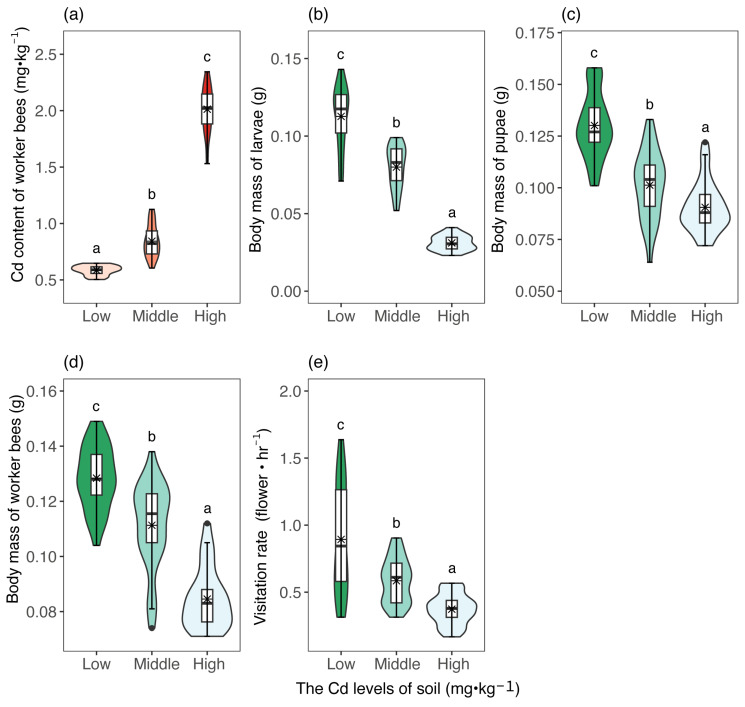
The violin plots show the amount of Cd in adult honey bees: (**a**), larva weight (**b**), pupa weight (**c**), adult honey bee weight (**d**), and visitation rates of honey bees (**e**) among the treatments. Violin plots show the density (width), interquartile range (hinges) and 1.5 times the interquartile range (adjacent lines). Line within the box represents the median of the responses and blue star within the box represents the mean of the responses. Different letters above the boxes denote significant differences among treatments (*p* < 0.05). Low, Middle, and High represent low, middle, high levels of soil Cd treatments, respectively.

Soil Cd concentration impacted the body mass of the larvae, pupae, and worker bees, as well as visitation rates of honey bees (Figure 1). The body mass of larvae, pupae, and worker bees showed a substantial decrease as the soil Cd concentration increased. The larvae, pupae, and worker bees experienced a decrease in body mass of 20.20%, 22.31%, and 13.28%, respectively, in the middle concentrations of soil Cd treatments. At high concentrations of soil Cd treatment, the bees’ body mass declined by 72.57%, 30.00%, and 35.94%. Furthermore, as the soil Cd concentration increased, there was a considerable rise in the Cd amounts of the worker bees, but a considerable decrease in visitation rates of honey bees.

### 3.3. The Relationships Among the Plant Traits, Honey Bee Growth and Foraging Behavior

**Figure 2:**
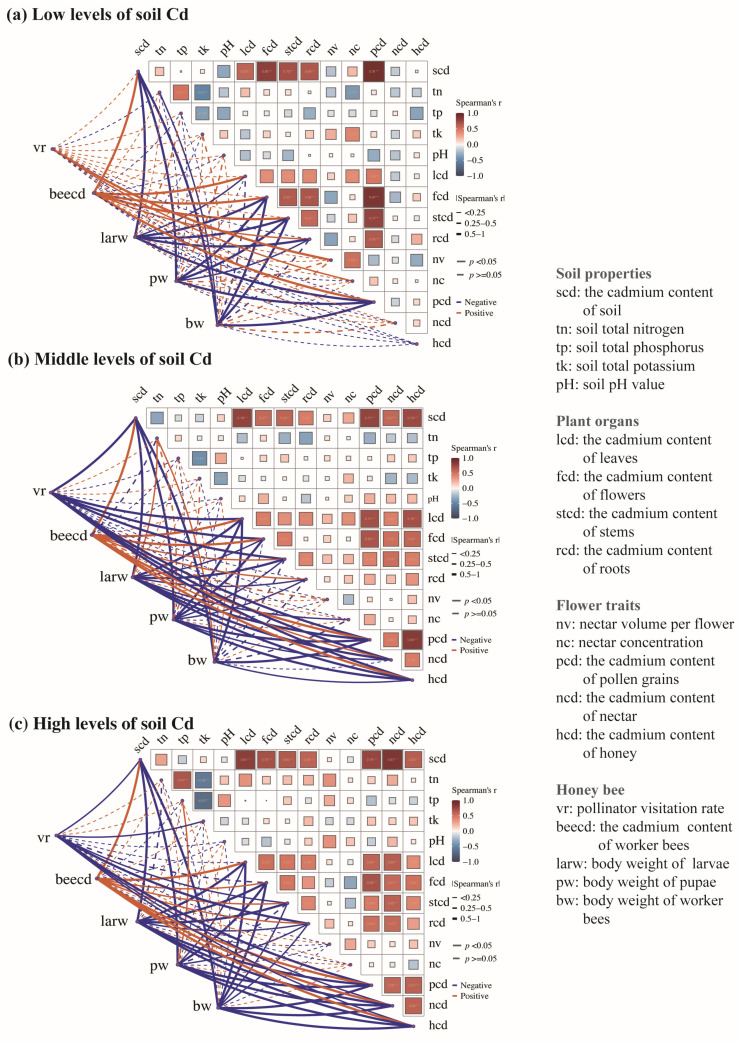
Spearman’s correlations among the plants, honey bees, and soil properties for the low (**a**), middle (**b**), and high (**c**) levels of soil Cd treatments. * *p* < 0.05, ** *p* < 0.01, *** *p* < 0.001. The red line represents a positive relationship, and the blue line represents a negative relationship. The square size represents the significant relationships among the variable. The different line widths represent the strengths of the relationships among the variables in pollinator attribution (body mass, Cd content of worker bees, and visitation rates) and the variations in the traits of plants, as well as the soil nutrition. The dotted lines represent the non-significant relationships among the variable.

The soil’s Cd level had a contrasting impact on the correlations between the traits (Figure 2). At the low concentrations of soil Cd treatments, we observed positive correlations between the concentration of Cd in the soil and the Cd amounts in several parts of the plant, including leaves, flowers, stems, and roots (*p* < 0.001, Figure 2a). There were positive associations between the Cd amounts of pollen and the Cd amounts of leaves, flowers, stems, and roots (*p* < 0.001). The visitation rates of honey bees showed a positive correlation with the volume of nectar per flower (*p* < 0.001). The amounts of Cd in worker bees directly correlate with the amounts of Cd in pollen, leaves, flowers, stems, and roots (*p* < 0.001). There was a negative correlation between larval, pupal, and worker bee body mass and the Cd content of pollen (*p* < 0.001).

At the middle and high concentrations of soil Cd treatments, the concentration of soil Cd showed positive correlations with the Cd amounts in the leaves, flowers, stems, and roots (*p* < 0.001, Figure 2b,c). The amounts of Cd in floral rewards (e.g., nectar and pollen) were found to be positively associated with the amount of Cd in leaves, flowers, stems, and roots (*p* < 0.001). There was a significant association between the Cd level in honey and nectar, as well as the Cd content in leaves, flowers, stems, and roots (*p* < 0.001). The visitation rates of honey bees showed a negative correlation with the amounts of Cd in nectar, honey, and pollen (*p* < 0.001). The amounts of Cd in worker bees showed a positive correlation with the amounts of Cd in nectar, honey, pollen, leaves, flowers, stems, and roots (*p* < 0.001). There was a negative correlation between the body mass of larvae, pupae, and worker bees and the Cd content of nectar, honey, and pollen (*p* < 0.001).

**Figure 3:**
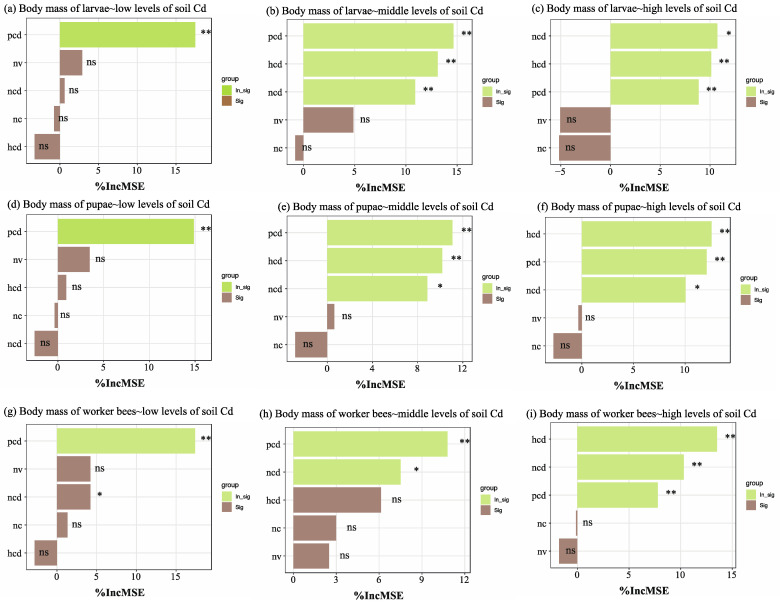
Random forest analysis identified food quality and quantity of honey bees as the factors most responsible for the changes in body weight. Body weight of larvae (**a**–**c**), pupae (**d**–**f**), and worker bees (**g**–**i**) at the low, middle and concentrations of soil Cd, respectively. % IncMSE indicates the increase in the Mean Squared Error when the given variable is randomly permuted. The larger the value of IncMSE, the greater the influence of the characteristic variable on the target. pcd: the Cd content of pollen; ncd: the Cd content of nectar; hcd: the Cd content of honey; nv: nectar volume per flower; nc: nectar concentration. ns: nonsignificant, * *p* < 0.05, ** *p* < 0.01.

At the low level of soil Cd treatments, the Cd content of pollen was the main factor that significantly affected the body mass of larvae, pupae, and worker bees (Figure 3, Figures S4 and S5). We also found that the amount of Cd in pollen, nectar, and honey significantly decreased the body mass of the larvae and pupae, particularly at the middle level of soil Cd treatments. The concentration of Cd in pollen and nectar, particularly under high levels of soil Cd treatments, primarily decreased the body mass of the adult honey bees. In addition, the amounts of Cd in pollen, nectar, and honey decreased the body mass of larvae, pupae, and worker bees when exposed to high levels of Cd in the soil.

Under low levels of soil Cd treatments, there was a direct correlation between the amount of Cd in pollen and the amount of Cd in worker bees (Figure S6). We found significant positive associations between the amounts of cadmium (Cd) in nectar, honey, and pollen and the amounts of Cd in worker bees across three treatments.

**Figure 4:**
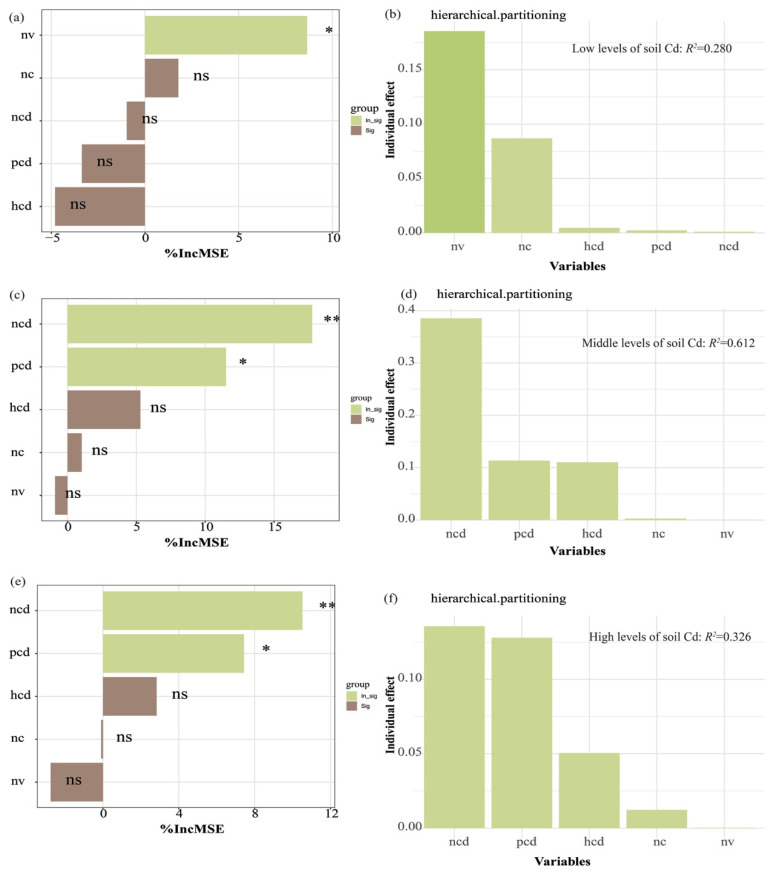
Random forest analysis identified food quality and quantity of honey bees as the factors most responsible for the changes in foraging behavior of honey bees (**a**,**c**,**e**). The relative importance of individual predictors in predicting visitation rates of honey bees was determined from fitting GLMMs (**b**,**d**,**f**). (**a**,**b**) represents the low concentrations of soil Cd treatments; (**c**,**d**) represents the middle concentrations of soil Cd treatments; (**e**,**f**) represents the high concentrations of soil Cd treatments. % IncMSE indicates the increase in the Mean Squared Error when the given variable is randomly permuted. The larger the value of IncMSE, the greater the influence of the characteristic variable on the target. pcd: the Cd content of pollen; ncd: the Cd content of nectar; hcd: the Cd content of honey; nv: nectar volume per flower; nc: nectar concentration. ns: nonsignificant, * *p* < 0.05, ** *p* < 0.01.

At low levels of soil Cd treatments, the nectar volume per flower strongly determined the visiting rates of honey bees (Figure 4 and Figure S7). There was a positive relationship between pollinator visitation rates and nectar volume per flower. Moreover, the presence of cadmium in pollen and nectar decreased the visitation rates of honey bees, particularly at moderate and high levels of soil cadmium treatments.

## 4. Discussion

Our findings indicated that soil cadmium significantly affected both the development and foraging behavior of honey bees. Honey bees exposed to low concentrations of soil cadmium exhibit a decrease in the body mass of larvae, pupae, and worker bees. This resulted from high levels of Cd in pollen. Additionally, soil cadmium modified the nectar amount per flower, hence affecting the foraging success of honey bees. A clear correlation existed between the number of floral visits and the nectar volume per flower. The presence of cadmium in pollen, nectar, and honey led to a reduction in the body mass of larvae, pupae, and worker bees in habitats with moderate and high soil cadmium levels. Moreover, the amount of cadmium in pollen and nectar influenced the frequency of honey bee visits to flowers. A significant negative connection existed between the Cd concentration in pollen and nectar and the number of floral visits. The data indicate that the specific relationships through which these effects transpired differ based on the dosage of soil cadmium.

The level of cadmium in pollen and nectar directly affects the richness, abundance, and foraging behavior of honey bees [12,17,19]. Research indicates a substantial correlation between soil cadmium concentration and the decline in both bee populations and diversity [6,18]. Moroń et al. [6] demonstrate a direct correlation between Cd levels in pollen and the reduction in bee abundance and diversity. Increased levels of Cd-contaminated pollen can impede larval development and disrupt the normal pupation process of bees [46]. Moreover, the presence of cadmium in nectar directly influences the growth and foraging behavior of bees [13,17]. Increased Cd concentrations in nectar extend developmental duration and diminish the body size of bees [19,47]. Our findings suggested that cadmium concentrations in nectar and pollen affected the growth and development of honey bees, while the specific impacts varied based on the concentration of soil cadmium. The exposure of honey bees to low levels of soil cadmium (Cd) revealed that the Cd concentration in pollen was the principal factor influencing honey bee body mass. As cadmium concentration in pollen increases, there is a notable decline in the body mass of larvae, pupae, and worker bees. A recent study has shown that honey bees may extract heavy metal ions from nectar [21]. In settings of low soil cadmium content, we observed that honey bees efficiently eliminated around 50% of cadmium from the nectar. The presence of cadmium in honey did not influence larval growth, as honey bees can remove cadmium ions from the honey. Nonetheless, the concentration of Cd in honey was markedly elevated in soil environments exhibiting moderate to high amounts of Cd. Borsuk et al. [21] assert that honey bees possess an optimal capacity for the removal of heavy metal ions. The quantities of cadmium in honey rose in the medium and high concentration treatments, and the cadmium levels in worker bees similarly elevated. We propose that the cadmium content in nectar inversely correlates with honey bees’ ability to remove cadmium. When a particular threshold is exceeded, the ability of bees to remove cadmium decreases. Thus, in soils with moderate to high Cd concentrations, the levels of Cd in nectar and pollen had a synergistic effect on the body mass of honey bees.

The quantity and quality of nectar and pollen significantly influence the foraging behavior of bees [30,48,49,50]. Increased levels of heavy metals in nectar and pollen reduce visitor frequencies [20]. Liu et al. [24] established that 4 mg·kg^−1^ Cd in soil resulted in a higher nectar yield and an enhanced rate of floral visits compared to the 8 mg·kg^−1^ Cd treatment. The nectar is hypothesized to include higher levels of Cd, resulting in a reduced frequency of honey bee visits to flowers. Xun et al. [13] found that the presence of heavy metals, such as copper, zinc, nickel, and lead, in nectar reduced the feeding duration of pollinators on specific flowers. The results demonstrate that the increasing concentration of soil cadmium is associated with a notable decline in the incidence of bees foraging on flowers. In our study, the factors affecting the frequency of honey bee visits to flowers fluctuated based on the Cd concentration in the soil. Low soil cadmium concentrations, on one hand, led to a single flower producing more nectar [24]. In contrast, honey bees may remove Cd [21], hence reducing the Cd concentration in nectar. The nectar quantity in each flower affected the frequency of honey bee visits when the soil exhibited low cadmium levels. As the plant grows in situations with moderate to elevated soil cadmium concentrations, the nectar volume per flower decreases. The concentration of cadmium in pollen and nectar increased concurrently. Nonetheless, honey bees often choose flowers with lower cadmium contents in their nectar and pollen [24]. Consequently, honey bees are compelled to reduce their floral visits and intentionally avoid collecting nectar with high cadmium contents.

The content of cadmium in pollen and nectar is correlated with its levels in various plant tissues [51,52]. The plant’s exposure to low cadmium (Cd) concentrations shows a positive correlation between Cd levels in pollen and those in the roots, stems, leaves, and flowers. The quantity of Cd in pollen, nectar, and honey exhibits a positive correlation with the Cd concentration in the roots, stems, leaves, and flowers of plants subjected to medium and high levels of Cd. A significant positive correlation was identified between Cd concentrations in roots, stems, leaves, and flowers and the Cd concentrations in the soil, regardless of treatment concentration. These findings indicate that the soil Cd permeates the aerial parts of plants through their roots, potentially disseminating to the stems and leaves, finally affecting the reproductive structures, including flowers. As a result, Cd accumulates in the nectar and pollen. The content of cadmium in pollen much exceeds that in nectar, suggesting that bees may remove Cd^2+^ from nectar [21].

The plasticity of floral traits influences pollinators’ foraging behavior and their selection of food resources [48]. Research indicates that soil heavy metals might alter the dimensions and abundance of flowers, thereby affecting the foraging behavior of pollinators [20]. Yet, we have not conducted any research on floral morphology at different Cd concentrations. Thus, it is unclear whether variations in flower size, plant height, and other characteristics influence the frequency of honey bee visits to the flowers. Moreover, our trials indicated a notable occurrence of dead honey bees around beehives located in environments with moderate to high concentration levels. The number of colonies was observed during the study to decrease by approximately one-third (observed by J. Liu). Therefore, more real-world evidence is needed to find out exactly how changes in cadmium content affect the death rate of honey bees. This project exclusively examined the mechanism by which different amounts of cadmium affect bee body mass and foraging behavior through field studies. However, it does not prove, through targeted feeding studies, how changing the amount of cadmium in nectar and pollen affects honey bees’ health, longevity, memory, and ability to think and reason, as well as their gut microbiome and their ability to reproduce and maintain a healthy population. Future endeavors necessitate increased empirical validation.

## 5. Conclusions

In conclusion, our results demonstrate that soil cadmium directly influences the body mass and foraging behavior of honey bees by altering the availability and nutritional quality of their food sources. It also indicates that the impact of soil cadmium on bee growth and foraging behavior is contingent upon the cadmium concentrations in the soil. These findings will offer new insights into the mechanisms influencing cadmium’s effects on pollinator development and foraging behavior, as well as the reasons for the worldwide decline of pollinators.

## Figures and Tables

**Table 1:** Effects of soil cadmium (Cd) content and soil nutrition on Cd content of leaves, flowers, stems and roots of *Brassica campestris* were analyzed using generalized linear mixed models (GLMMs).

	Cd Content of Leaves			Cd Content of Flowers			Cd Content of Stems			Cd Content of Roots		
Predictors	Estimates	CI	*p*	Estimates	CI	*p*	Estimates	CI	*p*	Estimates	CI	*p*
(Intercept)	0.16 ***	0.10–0.21	<0.001	−1.03 ***	−1.15–−0.91	<0.001	0.49 ***	0.46–0.53	<0.001	−0.46 ***	−0.60–−0.32	<0.001
tr [Cd_middle_]	0.19 ***	0.12–0.26	<0.001	0.38 ***	0.26–0.51	<0.001	1.55 ***	1.40–1.72	<0.001	0.45 ***	0.33–0.56	<0.001
tr [Cd_high_]	0.38 ***	0.31–0.44	<0.001	0.55 ***	0.43–0.67	<0.001	1.85 ***	1.66–2.06	<0.001	0.84 ***	0.73–0.95	<0.001
TN	0.02	−0.01–0.05	0.180	0.03	−0.02–0.08	0.277	1.00	0.96–1.05	0.939	0.01	−0.03–0.05	0.564
TP	−0.01	−0.04–0.02	0.448	0.00	−0.05–0.06	0.859	1.02	0.97–1.07	0.507	−0.02	−0.06–0.03	0.507
TK	−0.01	−0.04–0.02	0.583	0.02	−0.04–0.07	0.551	1.02	0.97–1.07	0.533	−0.00	−0.05–0.04	0.835
pH	−0.01	−0.03–0.01	0.401	−0.02	−0.06–0.03	0.440	0.97	0.93–1.01	0.131	−0.01	−0.05–0.02	0.470
Observations		90		90				90			90	
Marginal *R*^2^/Conditional *R*^2^		0.493/NA		0.808/0.859				0.659/NA			0.779/0.809	

Cd_middle_ and Cd_high_ refer to the middle and high levels of soil cadmium; TN, TP, TK and pH refer to total nitrogen, total phosphorus, total potassium, and pH of soils. *** p < 0.001.

**Table 2:** Effects of cadmium (Cd) and soil nutrition on the Cd content of pollen, nectar and honey of *Brassica campestris* were analyzed using generalized linear mixed models (GLMMs).

	Cd Content of Pollen			Cd Content of Nectar			Cd Content of Honey		
Predictors	Estimates	CI	*p*	Estimates	CI	*p*	Estimates	CI	*p*
(Intercept)	−2.21 ***	−2.30–−2.12	<0.001	−3.64 ***	−3.73–−3.55	<0.001	−4.36 ***	−4.50–−4.22	<0.001
tr [Cd_middle_]	0.41 ***	0.31–0.51	<0.001	0.23 ***	0.15–0.31	<0.001	1.19 ***	1.07–1.32	<0.001
tr [Cd_high_]	0.85 ***	0.76–0.94	<0.001	0.61 ***	0.54–0.69	<0.001	1.77 ***	1.65–1.89	<0.001
TN	0.00	−0.03–0.04	0.790	0.02	−0.01–0.05	0.207	0.00	−0.02–0.03	0.695
TP	−0.02	−0.06–0.02	0.281	−0.01	−0.05–0.02	0.425	−0.02	−0.05–0.00	0.090
TK	−0.01	−0.05–0.02	0.444	−0.00	−0.04–0.03	0.909	−0.00	−0.03–0.02	0.772
pH	−0.00	−0.03–0.03	0.800	0.01	−0.02–0.04	0.633	−0.01	−0.03–0.01	0.401
Observations	90			90			90		
Marginal *R*^2^/Conditional *R*^2^	0.984/0.995			0.961/1.000			0.996/1.000		

Cd_middle_ and Cd_high_ refer to the middle and high levels of soil cadmium; TN, TP, TK and pH refer to total nitrogen, total phosphorus, total potassium, and pH of soils. *** p < 0.001.

**Table 3:** Effects of cadmium (Cd) and soil nutrition on the nectar volume per flower and nectar concentration of *Brassica campestris* were analyzed using generalized linear mixed models (GLMMs).

	Nectar Volume per Flower			Nectar Concentration		
Predictors	Estimates	CI	*p*	Estimates	CI	*p*
(Intercept)	−0.11 **	−0.18–−0.04	0.002	3.76 ***	3.72–3.80	<0.001
tr [Cd_middle_]	−0.05	−0.10–0.01	0.097	−0.00	−0.06–0.06	0.897
tr [Cd_high_]	−0.20 ***	−0.26–−0.14	<0.001	0.04	−0.02–0.10	0.151
TN	−0.01	−0.03–0.02	0.600	−0.02	−0.05–0.01	0.144
TP	0.01	−0.02–0.04	0.456	0.02	−0.01–0.05	0.152
TK	0.01	−0.02–0.04	0.518	0.02	−0.00–0.05	0.102
pH	0.03 *	0.00–0.05	0.030	0.02	−0.01–0.04	0.181
Observations	90			90		
Marginal *R*^2^/Conditional *R*^2^	0.465/0.562			0.000/NA		

Cd_middle_ and Cd_high_ refer to the middle and high levels of soil cadmium; TN, TP, TK and pH refer to total nitrogen, total phosphorus, total potassium, and pH of soils. * p < 0.05, ** p < 0.01, *** p < 0.001.

## Data Availability

Data inquiries can be directed to the corresponding author.

## Supplementary Materials

The following supporting information can be downloaded at https://www.mdpi.com/article/10.3390/insects16030306/s1: Figure S1: The figure shows the study sites and the effects of soil cadmium on body weight and foraging behavior of honey bees; Figure S2: The violin plots show the amount of Cd in flower and leaf, soil pH, the amount of Cd in root, soil, and stem, total soil potassium, total soil nitrogen, and total soil phosphorus among the content of soil Cd treatments; Figure S3: The violin plots show the amount of Cd in pollen (a), nectar (b), and honey (c), as well as the nectar volume per flower (d), and nectar concentration (e) among the content of soil Cd treatments; Figure S4: The relative importance of individual predictors in predicting visitation rates of honey bees by glmm.hp; Figure S5: Correlation of body weight (larw, pw, and bw) and the amounts of Cd in honey bee’s food (pcd, ncd, hcd) among the variations in soil Cd treatments; Figure S6: Correlation of the amounts of Cd in adult honey bees (beecd) and the amounts of Cd in their food (pcd, ncd, hcd) among the variations in soil Cd treatments; Figure S7: Correlation of visitation rate of honey bees (vr) and the amounts of Cd in their food (pcd, ncd, hcd) among the variations in soil Cd treatments.
