# Supplementary material for: Cadmium Contaminants in Pollen and Nectar Are Variably Linked to the Growth and Foraging Behaviors of Honey Bees
Source: Insects. 2025 Mar 15;16(3):306. doi: 10.3390/insects16030306 (PMC11943015; doi:10.3390/insects16030306)
Supplement: Supplementary file 1 [file insects-16-00306-s001.zip › insects-3507775-supplementary.pdf]

Running head: soil cadmium impacts bee growth and foraging behavior

Cadmium contaminants in pollen and nectar are variably linked to the growth and foraging behaviors of honey bees

Dawei Li[1], Jia Liu[1], Yibin Yuan[2], Juanli Chen[1], Junpeng Mu[1]*

[1] Ecological Security and Protection Key Laboratory of Sichuan Province, Mianyang Normal University, Mianyang 621000, China

[2] Chengdu Academy of Environmental Science, Chengdu, 610072, China

*Correspondence: gbmujp@163.com

J Mu id: 0000-0001-5801-1878

Figure S1. The figure shows the study sites and the effects of soil Cadmium on body weight and foraging behavior of honey bees. There were three sites, and each site included three transects. The transect shows the difference in Cd concentration of soil, i.e., Low (the low concentration of soil Cd, which distance the 7 km form the electrical factors, and similarly for the other transects; grey ellipse), Middle (the middle concentration of soil Cd, which distance the 3 km form the electrical factors; light red ellipse), and High (the high concentration of soil Cd, which distance the 1 km form the electrical factors; dark red ellipse). nv: nectar volume per flower; nc: nectar concentration; ncd: the Cd content of nectar; pcd: the Cd content of pollen; hcd: the Cd content of honey; larw: the body mass of larvae; pw: the body mass of pupae; bw: the Cd content of worker bees; beecd: the Cd content in worker bees; vr: pollinator visitation rates. There were 10 plots for each treatment, The plot was 20 m × 15 m. Totally, there were 3 sites, 90 plots, and 90 hives.

Figure S2. The violin plots show the amount of Cd in flower and leaf, soil pH, the amount of Cd in root, soil, and stem, soil total potassium, soil total nitrogen, and soil total phosphorus among the content of soil Cd treatments. Violin plots show the density (width), interquartile range (hinges), and 1.5 times the interquartile range (adjacent lines). The line within the box represents the median of the responses, and the blue star within the box represents the mean of the responses. Different letters above the boxes denote significant differences among treatments ($p< 0.05$). Low, Middle, and High represent the low, middle, and high levels of soil Cd treatments, respectively.

Figure S3. The violin plots show the amount of Cd in pollen (a), nectar (b), and honey (c), as well as the nectar volume per flower (d), and nectar concentration (e) among the content of soil Cd treatments. Violin plots show the density (width), interquartile range (hinges), and 1.5 times the interquartile range (adjacent lines). The line within the box represents the median of the responses, and the blue star within the box represents the mean of the responses. Different letters above the boxes denote significant differences among treatments ($P < 0.05$). Low, Middle, and High represent the low, middle, and high levels of soil Cd treatments, respectively.

Figure S4. The relative importance of individual predictors in predicting visitation rates of honey bees determined from fitting GLMMs. Body weight of larvae (a-c), pupae (d-f), and adult honey bees (g-i) at the low, middle, and high concentrations of soil Cd, respectively. The Cd content of pollen is represented by pcd, the Cd content of nectar by ncd, the Cd content of honey by hcd, the nectar volume per flower by nv, and the nectar concentration by nc.

Figure S5. Correlation of body weight (larw, pw, and bw) and the amounts of Cd in honey bee’s food (pcd, ncd, hcd) among the variations in soil Cd treatments.

Figure S6. Correlation of the amounts of Cd in adult honey bees (beecd) and the amounts of Cd in their food (pcd, ncd, hcd) among the variations in soil Cd treatments. beecd: the Cd content of adult honey bees; pcd: the Cd content of pollen; ncd: the Cd content of nectar; hcd: the Cd content of honey. The color gradient represents the value

of Cd content in adult honey bees. Low, Middle, and High represent the low, middle, and high levels of soil Cd treatments, respectively.

Figure S7. Correlation of visitation rate of honey bees (vr) and the amounts of Cd in their food (pollen, nectar, honey) among the variations in soil Cd treatments.

Figure S1

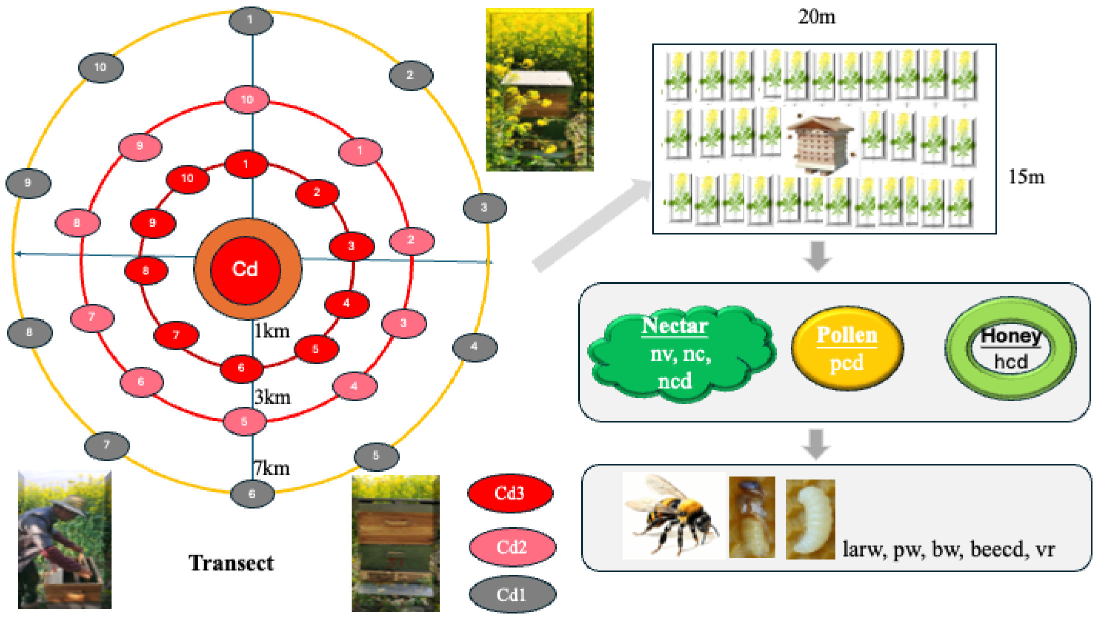
Cd
1km
3km
7km
Transect
Cd3
Cd2
Cd1
20m
15m
Nectar
nv, nc,
ncd
Pollen
pcd
Honey
hcd
larw, pw, bw, beecd, vr

Figure S2

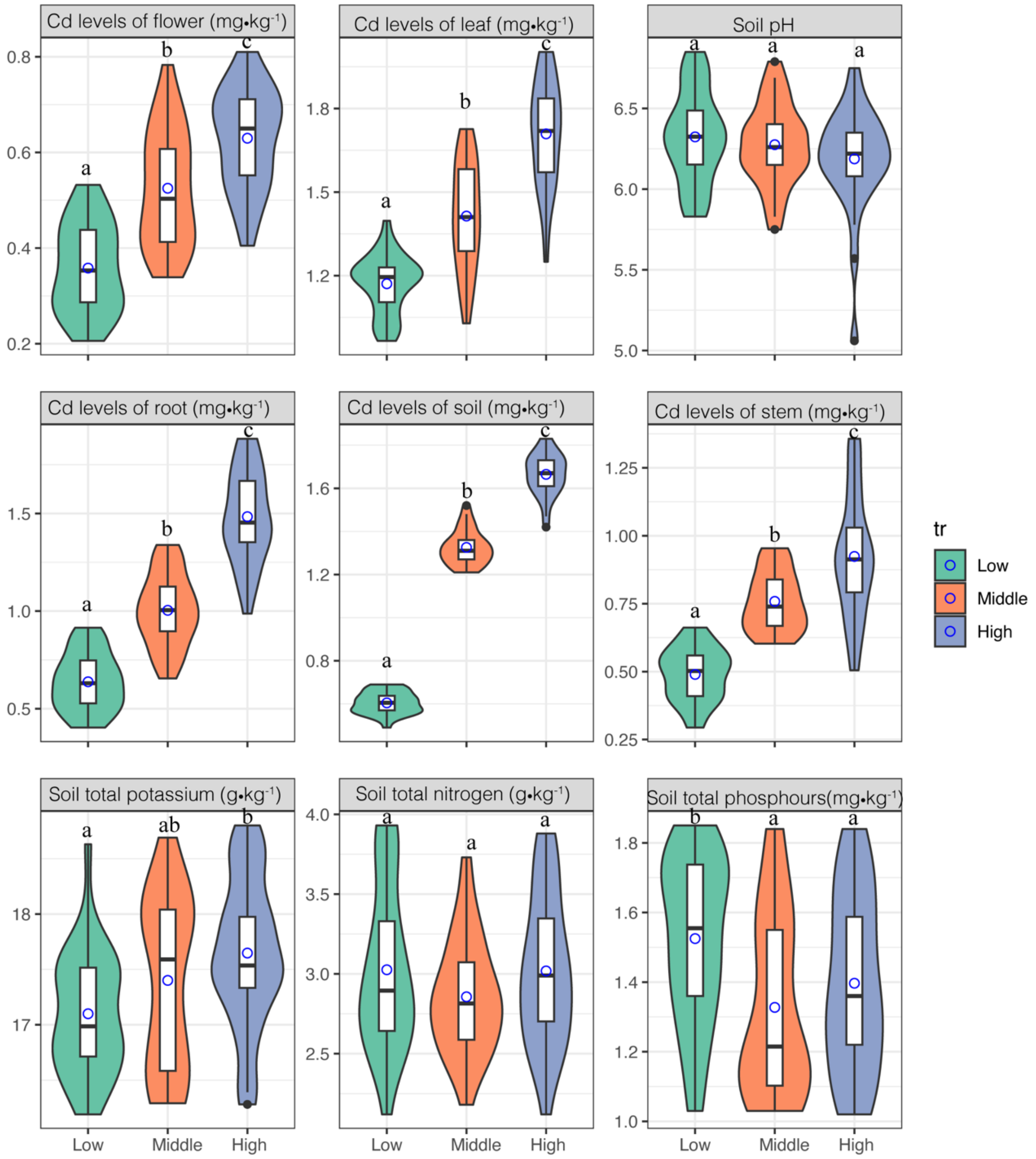

Cd levels of flower (mg•kg-1)
Cd levels of leaf (mg•kg-1)
Soil pH
Cd levels of root (mg•kg-1)
Cd levels of soil (mg•kg-1)
Cd levels of stem (mg•kg-1)
Soil total potassium (g•kg-1)
Soil total nitrogen (g•kg-1)
Soil total phosphours(mg•kg-1)
a
b
c
ab
Low
Middle
High
tr

Figure S3

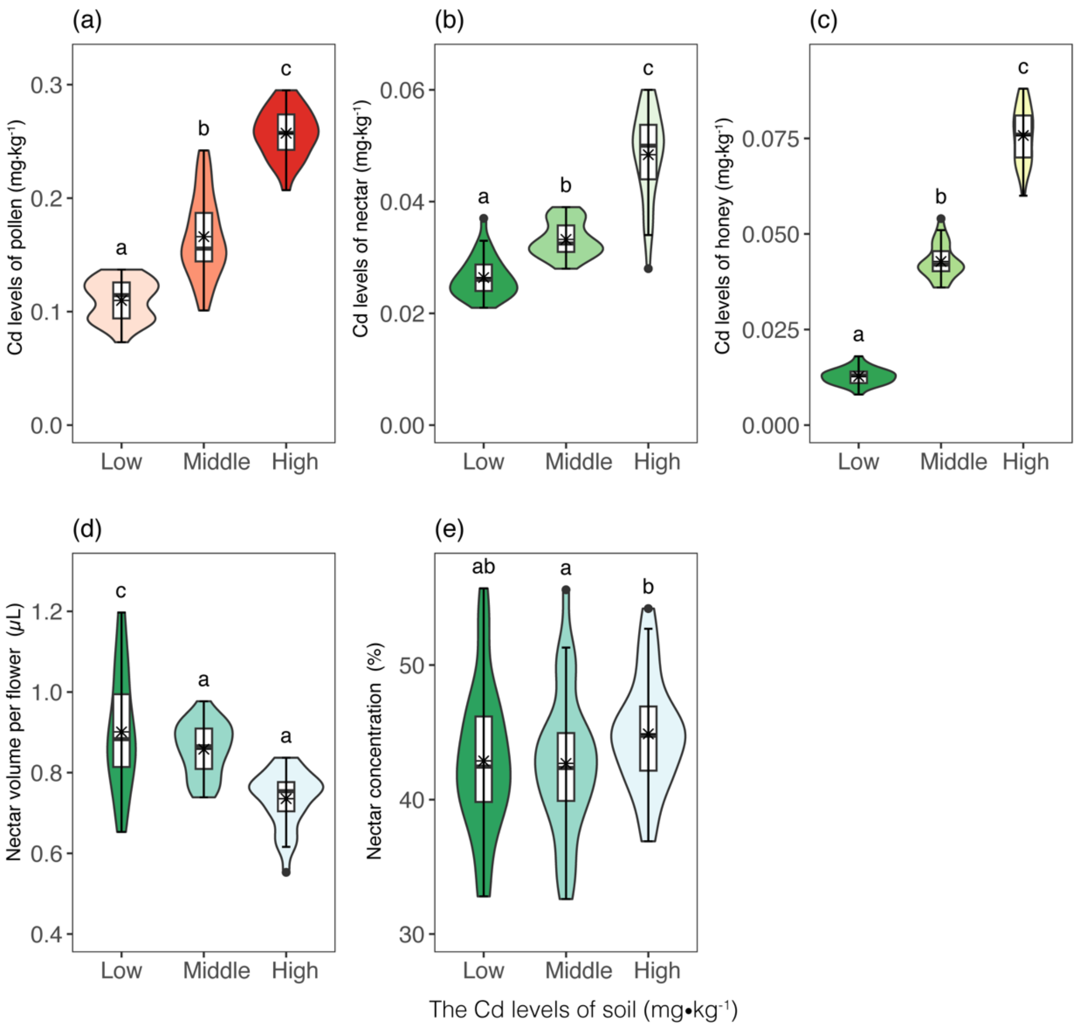

(a)
Cd levels of pollen (mg·kg-1)
0.0
0.1
0.2
0.3
a
b
c
Low
Middle
High
(b)
Cd levels of nectar (mg·kg-1)
0.00
0.02
0.04
0.06
a
b
c
Low
Middle
High
(c)
Cd levels of honey (mg·kg-1)
0.000
0.025
0.050
0.075
a
b
c
Low
Middle
High
(d)
Nectar volume per flower (μL)
0.4
0.6
0.8
1.0
1.2
c
a
a
Low
Middle
High
(e)
Nectar concentration (%)
30
40
50
ab
a
b
Low
Middle
High
The Cd levels of soil (mg•kg-1)

Figure S4

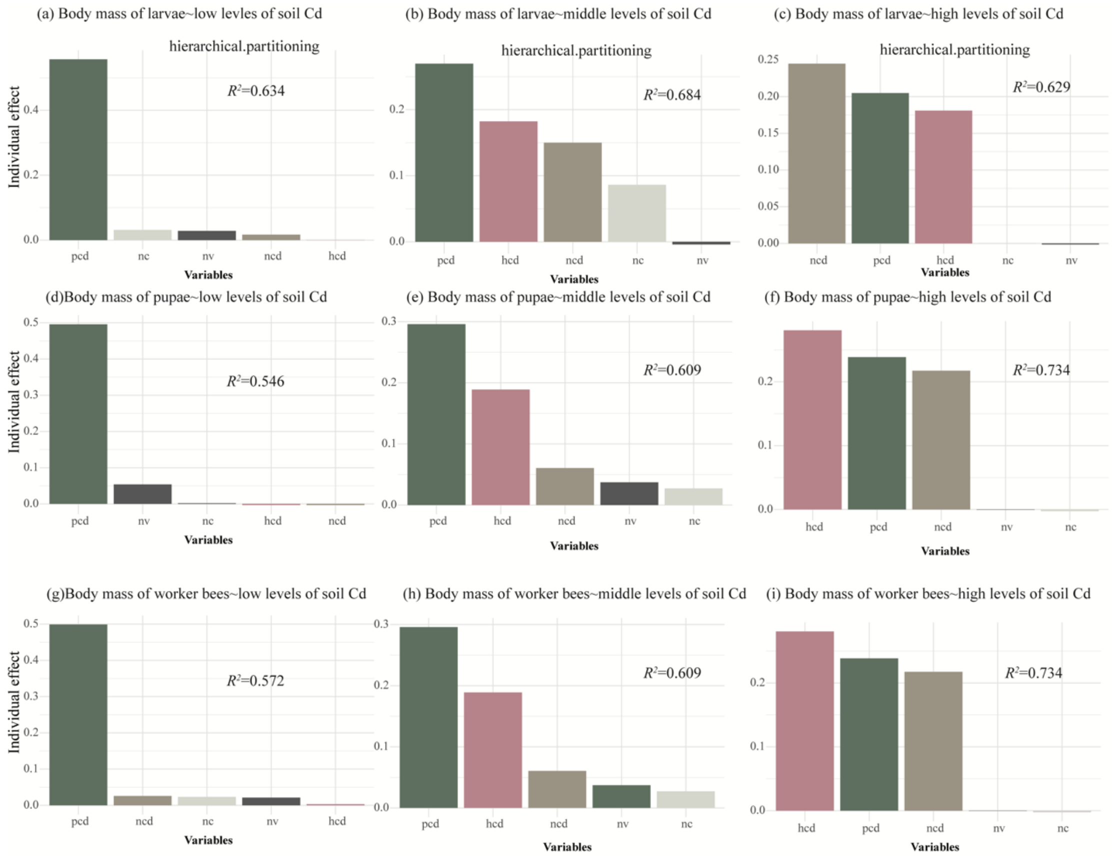
(a) Body mass of larvae~low levles of soil Cd
hierarchical.partitioning
R²=0.634
Individual effect
Variables
pcd nc nv ncd hcd
(b) Body mass of larvae~middle levels of soil Cd
hierarchical.partitioning
R²=0.684
Variables
pcd hcd ncd nc nv
(c) Body mass of larvae~high levels of soil Cd
hierarchical.partitioning
R²=0.629
Variables
ncd pcd hcd nc nv
(d)Body mass of pupae~low levels of soil Cd
R²=0.546
Individual effect
Variables
pcd nv nc hcd ncd
(e) Body mass of pupae~middle levels of soil Cd
R²=0.609
Variables
pcd hcd ncd nv nc
(f) Body mass of pupae~high levels of soil Cd
R²=0.734
Variables
hcd pcd ncd nv nc
(g)Body mass of worker bees~low levels of soil Cd
R²=0.572
Individual effect
Variables
pcd ncd nc nv hcd
(h) Body mass of worker bees~middle levels of soil Cd
R²=0.609
Variables
pcd hcd ncd nv nc
(i) Body mass of worker bees~high levels of soil Cd
R²=0.734
Variables
hcd pcd ncd nv nc

Figure S5

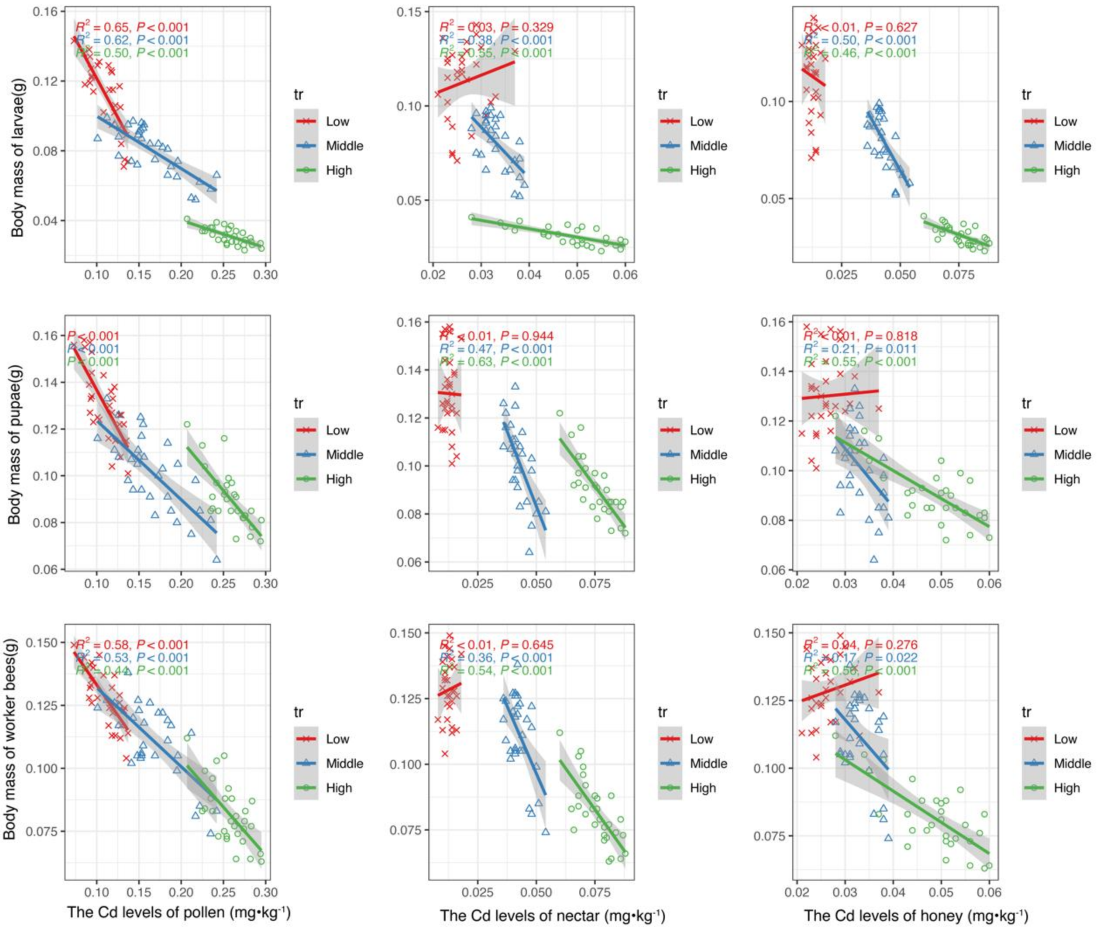

Body mass of larvae(g)
Body mass of pupae(g)
Body mass of worker bees(g)
The Cd levels of pollen (mg•kg⁻¹)
The Cd levels of nectar (mg•kg⁻¹)
The Cd levels of honey (mg•kg⁻¹)
tr
Low
Middle
High
R^2 = 0.65, P < 0.001
R^2 = 0.62, P < 0.001
R^2 = 0.50, P < 0.001
P = 0.329
P < 0.001
R^2 = 0.58, P < 0.001
R^2 = 0.53, P < 0.001

Figure S6

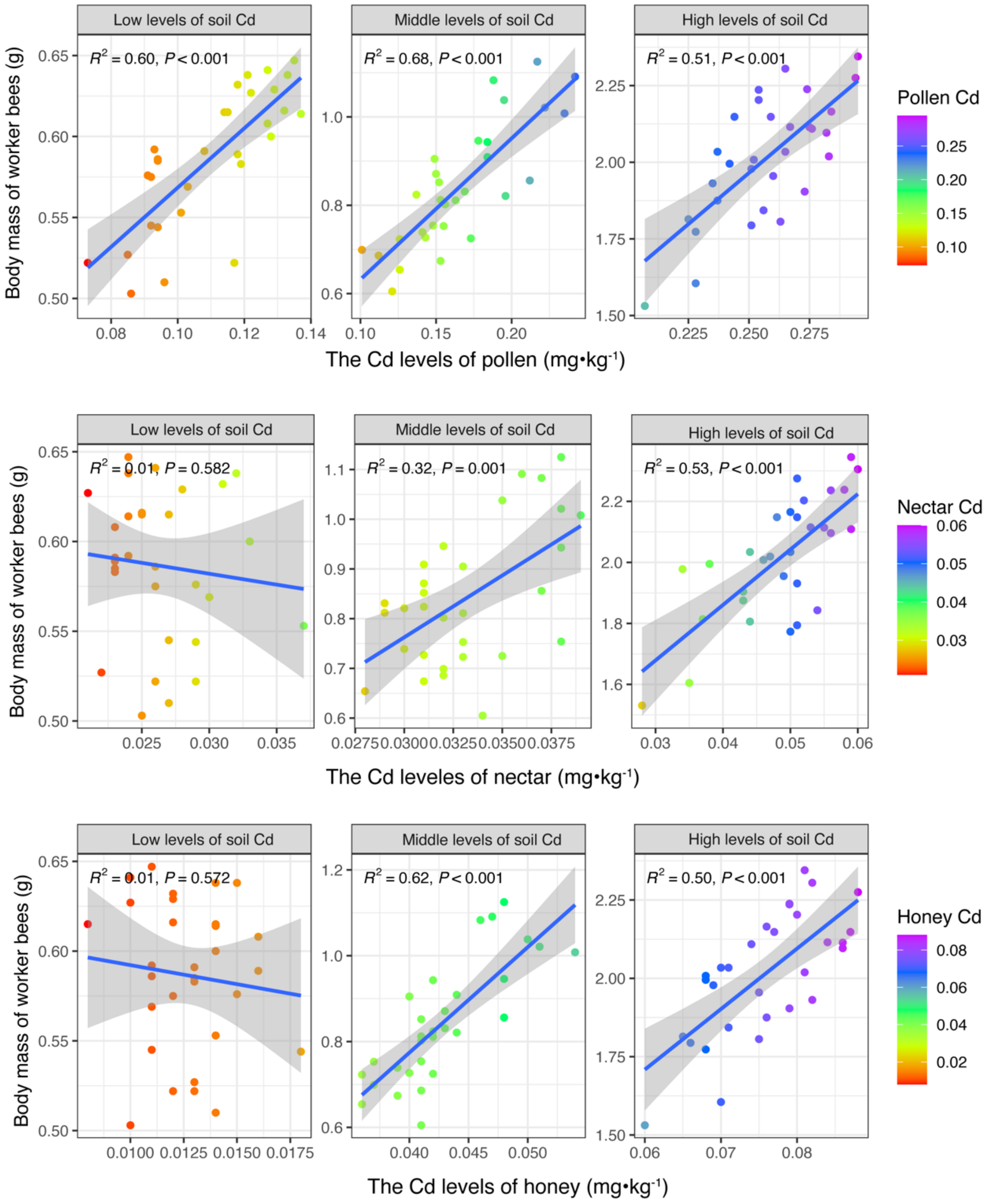

Low levels of soil Cd
Middle levels of soil Cd
High levels of soil Cd
R^2 = 0.60, P < 0.001
R^2 = 0.68, P < 0.001
R^2 = 0.51, P < 0.001
Body mass of worker bees (g)
The Cd levels of pollen (mg•kg-1)
Pollen Cd
R^2 = 0.01, P = 0.582
R^2 = 0.32, P = 0.001
R^2 = 0.53, P < 0.001
The Cd leveles of nectar (mg•kg-1)
Nectar Cd
R^2 = 0.01, P = 0.572
R^2 = 0.62, P < 0.001
R^2 = 0.50, P < 0.001
The Cd levels of honey (mg•kg-1)
Honey Cd

Figure S7

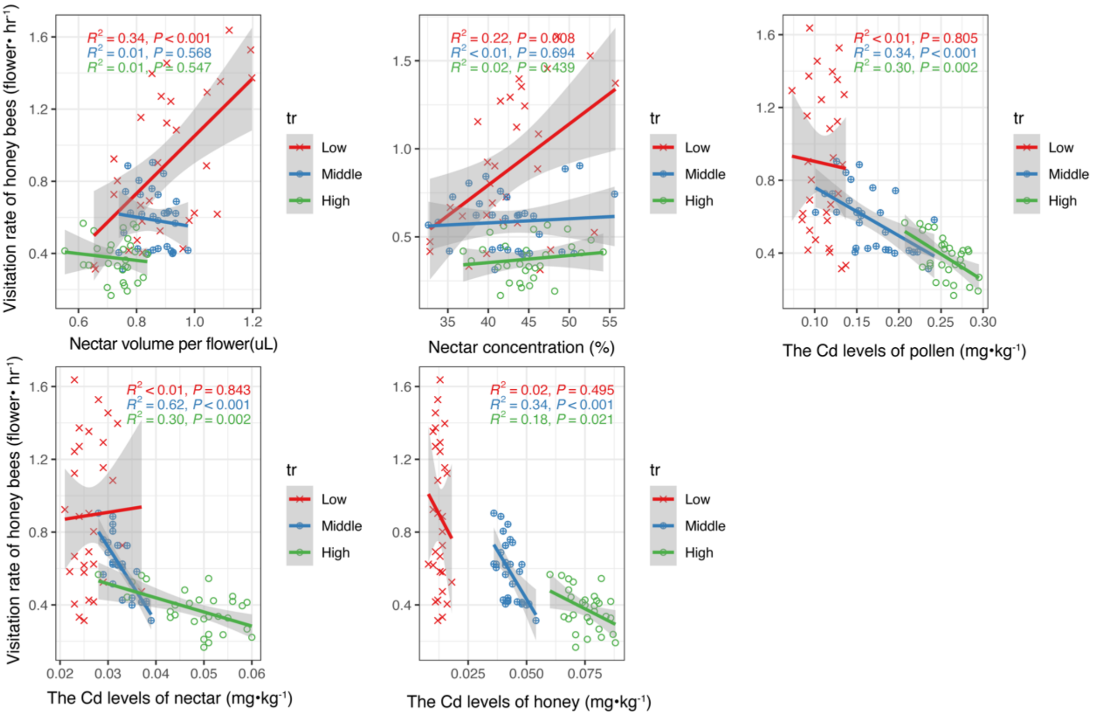

$R^2 = 0.34$, $P < 0.001$
$R^2 = 0.01$, $P = 0.568$
$R^2 = 0.01$, $P = 0.547$
Visitation rate of honey bees (flower• hr⁻¹)
Nectar volume per flower(uL)
$R^2 = 0.22$, $P = 0.008$
$R^2 < 0.01$, $P = 0.694$
$R^2 = 0.02$, $P = 0.439$
Nectar concentration (%)
$R^2 < 0.01$, $P = 0.805$
$R^2 = 0.34$, $P < 0.001$
$R^2 = 0.30$, $P = 0.002$
The Cd levels of pollen (mg•kg⁻¹)
$R^2 < 0.01$, $P = 0.843$
$R^2 = 0.62$, $P < 0.001$
$R^2 = 0.30$, $P = 0.002$
The Cd levels of nectar (mg•kg⁻¹)
$R^2 = 0.02$, $P = 0.495$
$R^2 = 0.34$, $P < 0.001$
$R^2 = 0.18$, $P = 0.021$
The Cd levels of honey (mg•kg⁻¹)
tr
Low
Middle
High
